# Conservative Management of a Delayed Benign Gastrobronchial Fistula: A 20-Year Follow-up

**DOI:** 10.7759/cureus.5444

**Published:** 2019-08-20

**Authors:** Mohammadali M Shoja, Khalil Ansarin

**Affiliations:** 1 Surgery, University of Texas Medical Branch, Galveston, USA; 2 Internal Medicine, Tabriz University of Medical Sciences, Tabriz, IRN

**Keywords:** fistula, gunshot wound, lung, stomach

## Abstract

We describe a case of gastrobronchial fistula (GBF) following a thoracoabdominal gunshot wound in a previously healthy young man. Despite initial surgery, the patient suffered recurrent hemoptysis, and a GBF was diagnosed 18 months after initial presentation. The patient was treated with oral proton pump inhibitors for a prolonged period with the resolution of the fistula. During a follow-up 20 years later, no recurrence of the fistula was noted. The importance of early diagnosis of such fistulae cannot be overstated. This report provides a testimony to the feasibility of the conservative approaches in managing delayed, benign, and post-traumatic GBF.

## Introduction

Ever since John Hunter’s celebrated *Treatise on the Blood, Inflammation and Gunshot Wounds* was published in 1794, the surgical and other sequelae of gunshot wounds have been matters of great clinical interest. Among the numerous publications arising from the treatment and management of gunshot victims are some that describe the formation of fistulae following wounds to the thorax and abdomen. These include cases of vascular injury resulting in aortoenteric fistula, but most concern fistulae between the respiratory and alimentary tracts. In at least one case, such a fistula was not diagnosed until long after the shooting accident [1]. In this article, we describe a patient hospitalized after a gunshot wound that penetrated the thorax and upper abdomen, leading to a long succession of clinically serious consequences requiring various approaches to management. The clinical course was complicated by the formation of gastrobronchial fistula (GBF), which was successfully managed by prolonged anti-acid therapy.

## Case presentation

A previously healthy 25-year-old man was admitted to our hospital after a gunshot wound on May 1987. The bullet had entered the left side of his back at the 10th intercostal space and passed upward and laterally to exit at the fifth intercostal space three centimeters medial to the anterior axillary line. The patient had sustained colonic perforation, left diaphragmatic, splenic and lung lacerations and left bronchial rupture. A left thoracostomy tube was inserted and the gunshot entrance and exit wounds were sealed. While supportive therapy was ongoing, he developed tachycardia and fever and was found to have diminished breath sounds and dullness to percussion over the left lower thorax. A chest X-ray revealed left pleural effusion, which proved to be an empyema. With suspicion of esophageal perforation, he was taken to the operating room on the third day of admission. After exploration, fecal material and displaced stomach were noted in the left chest cavity. The lacerated diaphragm was repaired, and a loop colostomy was performed. A splenectomy was also performed due to the splenic laceration.

Two weeks after the operation, there was a significant bleeding episode through the left chest tube. A chest X-ray revealed left lung collapse and left pneumothorax and hemothorax. Upper gastrointestinal endoscopy and bronchoscopy did not reveal the bleeding site. Fiberoptic bronchoscopy revealed thick yellowish secretions in the left lower lobe bronchus. The chest bleeding stopped spontaneously. Later, a foul-smelling greenish secretion appeared in the chest tube and continued for two months. Aerobic culture of the secretion revealed Klebsiella, Escherichia coli and more than 15 species of Gram-positive bacteria (due to contamination). The patient was treated with a variety of antibiotics. Two months later, a window for drainage of the secretion was made on the left hemithorax at the eighth intercostal space. A second bronchoscopy was unremarkable. Patient’s general condition was improved but drainage of the purulent secretions from the left thorax continued for another month. The patient was discharged in a stable condition six months after the initial admission.

**Figure 1:**
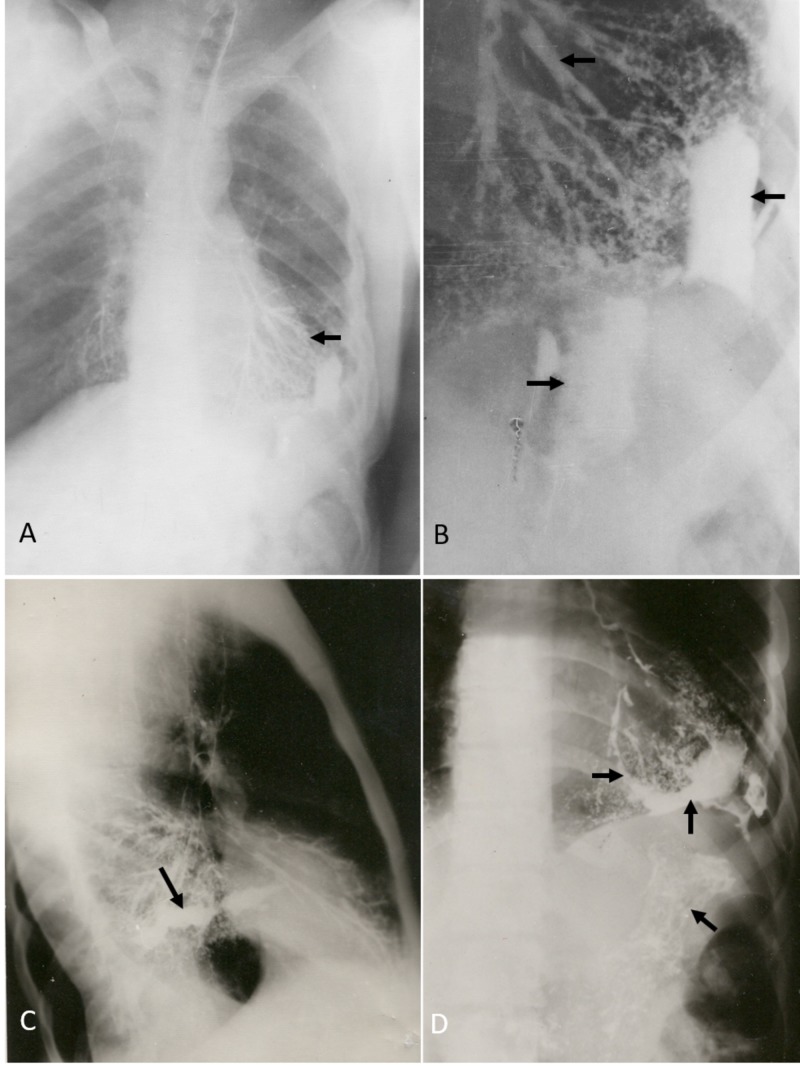
The appearance of a gastrobronchial fistula in bronchography The bronchogram shows the contrast material (arrows) progresses from the left bronchial tract (the arrow in A and the upper arrow in B) to the pleural cavity (the middle arrow in B and the arrow in C) and enters the stomach (the lower arrow in D). A, B and D are the anteroposterior chest views and C is the lateral view.

In the months following discharge, the patient had cough, hemoptysis, epigastric pain, vomiting, dysuria, hematuria and left costovertebral angle tenderness. A repeat bronchoscopy revealed no discernible pathology. The repeat upper gastrointestinal endoscopy reported two small ulcers in the lesser curvature and the duodenal bulla as well as bile reflux, which were treated with appropriate medications. A non-opaque renal stone was noted on radiology. Intravenous pyelography further showed a distorted lower renal pole and ultrasound examination revealed a decreased echogenicity at the same area. Urine exam revealed microscopic proteinuria but was negative for acid-fast bacilli. One month later, a bronchography was performed owing to continued hemoptysis. This revealed the leakage of the contrast material into the left pleural cavity through a communicating port from a branch of the lateral segmental bronchus of the left lower lobe and progression of the contrast down to the stomach (Figure 1). A diagnosis of GBF was made. A surgical repair was under consideration, but the patient’s condition improved with antacids treatment administered for a prolong period. Hemoptysis and melena gradually ceased without any significant pulmonary complications. In a 20-year follow-up, the patient had no recurrence of fistula. He was intermittently suffering from urolithiasis, urethral stenosis, occasional dysphagia, esophageal hiatal hernia, and intestinal adhesions.

## Discussion

Gastrobronchial and similar fistulae arising under various conditions have been described in the literature. For example, such fistulae have been reported as a complication of posterolateral hernia of the diaphragm, as an iatrogenic consequence of esophageal perforation during tracheotomy or use of a feeding tube, following esophagectomies, partial esophageal resections, gastrectomy, splenectomy or gastroplasty for obesity, as a sequela of esophageal carcinoma, and as a complication of recurrent pancreatitis, subphrenic abscess, neoplastic disorders, penetration trauma or perforated peptic ulcer [2-11]. One recognized cause is gunshot wounds to the thorax that may also penetrate the upper abdomen. Recurrent infections and the abdominal-thoracic pressure difference contribute to the formation and persistence of fistulous connections between the stomach and bronchial tree [1].

Fistulae between the respiratory and gastrointestinal tracts can simulate bronchiectasis and result in recurrent episodes of inflammatory or infectious conditions [12]. Chronic cough and respiratory difficulty are common symptoms, and lung or subphrenic abscess formation a common complication. Pleural involvement can result in effusion, empyema or pneumothorax [10,13]. Acute respiratory distress syndrome is a rare complication of GBF. It has been reported that a bronchial adenocarcinoma may arise from an untreated, benign GBF [14]. The diagnosis of GBF is now made by a contrast-enhanced computed tomography (CT) scan [12]. Historically, bronchography and fistulogram was the gold-standard diagnostic test but is now infrequently performed due to increasingly improved resolution of the current CT imaging. 

Surgical resection of GBF and the adjacent lung lobe along with the diaphragmatic repair is often considered the definitive treatment for GBF. Hitchcock and colleagues surgically treated a patient with GBF secondary to a gastrodiaphragmatic gunshot [1]. The endoscopic closure of the GBF using clip and temporary stent has been shown to result in complete closure of fistula within 12 weeks and is suggested by some as the first-line treatment [15,16]. However, surgery is seldom or never an emergency [12]. Some patients have been successfully managed by conservative approaches. Martin-Smith and colleagues treated a patient with post-esophagectomy GBF with enteral nutrition, antibiotics, and respiratory support [17]. Matsuoka and colleagues reported a patient with gastric ulcer penetrating the liver and forming a GBF who underwent a total gastrectomy for massive gastric bleeding; however, these authors maintained that a conservative management with anti-acids should be the initial therapeutic option, but this should, of course, be attempted in the absence of a serious complication necessitating a surgery [18]. Sakran and colleagues successfully employed a nonoperative treatment with aggressive drainage regimens and nasojejunal feeding in three of six patients with GBF [13]. A delayed diagnosis of fistula may allow epithelialization and persistence of the fistulous tract and may theoretically diminish the success of conservative management [17]. Our patient presented with a delayed GBF (18 months after initial injury) and conservative management proved to succeed. In a 20-year follow-up, no recurrence of the fistula was noted.

## Conclusions

Clinicians should be aware of the delayed complications of thoracoabdominal penetrating wounds, such as fistula formation. The management of GBF is controversial owing to a paucity of the literature. The treatment choice is usually tailored to the complexity and severity of the fistula, underlying cause, patient’s comorbidities, and managing physician’s experience. In the trauma-related GBF, a conservative approach to treatment seems to be an effective measure and, in our experience, it can be safely tried before any endoscopic or surgical measures. The minimally-invasive (such as endoscopic clipping/stenting) or surgical approaches should be preserved for refractory cases or patients with other complications necessitating a surgery. Aside from anti-acid treatment, proper nutrition and pulmonary toileting are cornerstones of conservative management. Delayed cases of GBF are often difficult to resolve conservatively; however, even in such delayed cases, conservative management could still be attempted first if the patient's condition allows that. The present report adds testimony to the feasibility of conservative, anti-acid therapy in the treatment of delayed, benign, and post-traumatic GBF.
